# Beneficial effect of *Boswellia serrata* gum resin on spatial learning and the dendritic tree of dentate gyrus granule cells in aged rats

**Published:** 2016

**Authors:** Mohammad Hosseini-Sharifabad, Razieh Kamali-Ardakani, Ali Hosseini-Sharifabad

**Affiliations:** *Department of Biology and Anatomical Sciences, Shahid Sadoughi University of Medical Sciences, Yazd, Iran*

**Keywords:** *Aging*, *Boswellia serrate*, *Dendrite*, *Dentate gyrus*, *Spatial learning*

## Abstract

**Objective:**

The hippocampal formation, particularly the dentate gyrus (DG), shows age-related morphological changes that could cause memory decline. It is indicated that Boswellia resins attenuates memory deficits and the major component of *Boswellia serrata* (Bs) gum resin, beta boswellic acid increased neurite outgrowth and branching in hippocampal neurons. This study was designed to investigate the effect of Boswellia treatment on spatial learning performance and the morphology of dentate granule cells in aged rats.

**Materials and Methods:**

Sixteen male Wistar rats (24 months old) were divided into experimental and control groups. Experimental group was intragastrically administered with the aqueous extract of Bs (100 mg/kg/d for 8 weeks) and control group received a similar volume of water. Spatial learning performance of rats was tested using Morris water maze task. At the end of experiment, the brain was removed and the right hippocampus was serially sectioned for morphometric analysis. The Cavalieri principle was employed to estimate the volume of the DG. A quantitative Golgi study was used to analyze the dendritic trees of dentate granule cells.

**Results:**

Chronic treatment with Bs improved spatial learning capability during the three acquisition days. Comparisons also revealed that Bs-treated aged rat had greater DG with increased dendritic complexity in the dentate granule cells than control rats. Hippocampal granule cells of Bs-treated aged rats had more dendritic segments, larger arbors, more numerical branching density and more dendritic spines in comparison to control animals.

**Conclusion:**

This study provided a neuro-anatomical basis for memory improvement due to chronic treatment with Bs.

## Introduction

Maintaining brain health throughout the life, particularly during aging when neurodegenerative diseases like Alzheimer’s disease (AD) occur, is an important public health goal. It is well known that the hippocampal dendritic systems, as the functional core of neuronal ensembles, play a critical role in spatial learning and memory (Rusakov et al., 1997 ▶). The hippocampal formation and specially the dentate gyrus (DG) have been the focus of studies investigating the neuro-biological cause of age-related memory decline. The DG, as the gateway to the hippocampus is the main receptive field for afferent projections from neocortical areas (Scharfman, 2007 ▶). The reductions in the dendritic arborization (Geinisman et al., 1978 ▶; Geinisman et al., 1977 ▶; Luebke et al., 2003 ▶; Von Bohlen et al., 2006 ▶), synaptic spine loss (Geinisman et al., 1992 ▶) was observed in the dentate granule cells of aged animals. 

The use of plant extracts in the treatment of diseases is millennia old and continues to date. Gum resin of the trees of the *Boswellia* species from Burseraceae family, commonly called Olibanum or Frankincense, has been traditionally used in the treatment of various chronic inflammatory diseases (Duwiejua et al., 1993 ▶, Moussaieff and Mechoulam 2009 ▶; Safayhi and Ammon, 1997 ▶). The four main species producing this resin are *Boswellia carteri* (East Africa), *Boswellia papyrifera* (Eritrea), *Boswellia sacra* (southern Arabia) and *Boswellia serrata* (north-western India).

In Iran, it is used to prevent amnesia and enhance memory as recommended by the Persian physician, Avicenna, in The Canon of Medicine (The Law of Medicine), in the 10th century. Currently, the beneficial effects of Boswellia in improvement of learning and memory in young and adult animals is well documented by Iranian researchers (Farshchi et al., 2010 ▶; Hosseini et al., 2010 ▶; Mahmoudi et al., 2011 ▶). However, the effect of *Boswellia serrata* (Bs) on the learning and memory in aged animals are still poorly understood. *In vitro* studies have reported that the major component of Bs gum resin, beta boswellic acid, increased neurite outgrowth and branching in hippocampal neurons (Karima et al., 2010 ▶). It is also known that the hippocampal dentate granule cells shows remarkable plasticity including remodeling and synaptic turnover (Cameron and Gould, 1996 ▶). On the basis of this background, this study was designed to experimentally investigate the effects of chronic administration of Bs on the learning performance and the morphology of hippocampal granule cells in aged rat. In the present study, spatial learning and memory performance were evaluated using Morris water maze (Morris, 1984 ▶). Cavalieri principle (Gundersen et al., 1988 ▶), was employed to estimate the volumes of constituents layers of DG and a quantitative Golgi study was used to analyze dendritic branches of dentate granule cells

## Materials and Methods

**Animals and treatment**

Male albino Wistar rats aged 24 months (570–600 g), obtained from the animal house of Isfahan Medical Faculty, Iran, were housed in pairs and kept under standard laboratory conditions with food and water available *ad libitum* and a 12:12 light-dark cycle (lights on at 7:00 am). The animals were randomly divided into experimental and control groups (n=8). The animals in experimental group received 100 mg/kg per day Bs gum resin orally for 8 weeks. The control group, was administered with a similar volume of water. The Boswellia gum resin with certified botanical origin, *Boswellia serrata *Roxb, was received as a gift from Goldarou phytolaboratory (Isfahan, Iran). 

This powdered extract was dissolved in water, then diluted with water to the desired concentration and administered via oral feeding needle at a volume of 5 ml/kg body weight.

All animal experiments and housing was performed in accordance with rules approved by the Ethical Committee of Shahid Sadoughi Medical University, Iran.

**Water maze test**

Morris water maze (Morris, 1984 ▶) consisted of a black circular pool (diameter: 180 cm, height: 60 cm) filled up to 30 cm with water at room temperature. Animals that were placed in the water were able to escape only by finding a black platform (12 × 12 cm), which was hidden 1 cm under the surface in the centre of one of the quadrants of the maze. Finding the platform within 120 s was the animals' challenge. In case of failure within the mentioned period, the animal was gently guided to the platform by hand. Each rat received three acquisition trials per day for 3 days. The time (in seconds) spent from placing the animal into the water until finding the platform (escape latency) and the swimming speed were recorded for each trial. The length of swim path (distance traveled) was also measured, but not used for data analysis, because it correlated with escape latency measurements. 

After swimming, the rats were allowed to stay on the platform for 30 s. Animals were given 20 min rests between trials. Behavior in the maze was recorded by a video-tracking computer system (EthoVision, Noldus, The Netherlands).

**Histological procedure**

At the end of the experiment period, all rats were deeply anesthetized intraperitoneally with urethan (Merk, Germany) and transcardially perfused with a phosphate-buffered solution of 4% formaldehyde and 1% glutaraldehyde. The brains were removed from the skull, weighed and divided in the midsagittal direction. Right hemisphere was selected for morphometeric analysis of the DG. Brains were coded so that the investigators were blind to specimens. Each hemisphere was placed in a chilled slicing box and cut 6 mm from the front. The posterior portion of hemisphere which contained hippocampus was serially sectioned in a coronal plane at 100 µm with a calibrated vibratome (Diapath, Italy) and the sections were collected along the entire extent of the hippocampus. 

**Stereology**

To estimate the volumes of the layers of the DG, starting at a random position, every 5th section with an interval of 500 μm was taken for staining. The sections were mounted on gelatin-coated object glasses and dried at room temperature. The dry sections were stained using hematoxylin: dipped in distilled water, 4 minutes in hematoxylin, washed in running tap water for 10 minutes, rinsed with distilled water, dehydrated in 70% (10 minutes), 96% (2 5 minutes) and 99% ethanol (2 8 minutes), cleared 15 minutes in xylene. Finally, cover glasses were mounted. 

The DG is an easily recognized region due to intense staining and because it is not continuous with other hippocampal regions. The granular cell layer, as well as the principal cell layer of the DG, is composed of the smallest neuronal cell bodies and is the most densely packed layer in the hippocampus. The volumes of the constituent layers of the DG, i.e. the molecular, granular and the polymorphic (hilus) layers were estimated on the basis of the Cavalieri principle (Gundersen et al., 1988 ▶). The cross sectional areas of the layers of the DG were estimated by point-counting principle with a projection microscope (Zeiss, Germany) using a 4x objective lens at a final magnification of 87.5x. Each image was superimposed at random, with a grid of systematic uniform test points 10 mm apart. Each point represented an area, a (p) =0.013 mm^2^, in the section plane.

The number of points hitting the layers (P) was multiplied by the area associated with each point, a (p), to obtain an unbiased estimate of sectional area of each profile. The sum of sectional areas of each layer was used to estimate reference volume, V (ref), from the following relationship, where t represents the intersection distance; 

V (ref) = t. P. a( p) = t. A

No areal shrinkage correction was used in this study because no difference in shrinkage was found between groups (Mean areal shrinkage of 4% was detected).

**Morphometry**

Ten adjacent sections from the midpoint of septotemporal extent of the hippocampus were selected. They were processed according to a modified version of the single-section Golgi impregnation procedure (Gabbott and Somogyi, 1994 ▶). Briefly, the sections were incubated in 3% potassium dichromate in distilled water overnight, rinsed in distilled water, and mounted on plain slides and finally a coverslip was glued over the sections at four corners. They were incubated in 1.5% silver nitrate in distilled water overnight in darkness. On the following day, the slide assemblies were dismantled. Then, tissue sections were rinsed in distilled water and dehydrated in 95% ethanol followed by absolute ethanol. The sections were then cleared in xylene, mounted onto gelatinized slides and coverslipped under Permount (Fisher Scientific, Pittsburgh, PA, USA). 

From each histological section, one granule cell was selected at two-third the depth of suprapyramidal (upper) blade of the dentate granular layer. The morphological criteria used for selecting the neurons to be measured were similar to those used by De Ruiter and Uylings (1987 ▶). 

We observed an average of 7% shrinkage in the sections, with no difference between Bs–treated and control rats. These values were employed as a correction factor for the length measurements.

The presence of cut terminal segments on a neuron was not considered as a criterion for its exclusion from the estimations because the elimination of these neurons would have biased the sample towards smaller neurons (Uylings and Van Pelt, 2002 ▶). The dendritic trees of dentate granule cells were traced by hand with the aid of a camera lucida (LeitzOrthoplan, Wetzlar, Germany), at a final magnification of 640x. The centrifugal ordering of dendritic trees was used to estimate the number of dendritic segments per cell (Uemura et al., 1995 ▶). The branching density of dendritic trees was evaluated by applying the concentric circle method. A grid of concentric rings (successively 20 µm apart) was placed over the camera lucida drawing of the dendritic field, centered in the cell body, and the number of dendritic intersections crossing each concentric ring was counted. To measure the number of dendritic spines, 10 cells located in the inner and outer third of the DG molecular layer were analyzed per animal. In each cell, spine density per 20-µm segments of the proximal, middle and distal parts of the largest dendrite within the plane of section was analyzed. No distinction was made between the different morphological types of spines.

For metric analysis, the dendritic length was measured using a Zeiss interactive digitizing analysis system (Zeiss, Germany).

**Statistical analysis**

Student’s t-test was employed to compare data from the experimental and control rats. A difference was considered significant if p< 0.05. To prevent erroneous skewing of the results by subjects, morphological data for each rat was taken as the mean of the data for all cells recorded in the subject.

## Results

The body weight at the time of sacrifice did not differ between the control and Bs-treated rats (636±29.7 g for control and 628±26.2 g for Bs-treated, p=0.52)

**Figure 1:**
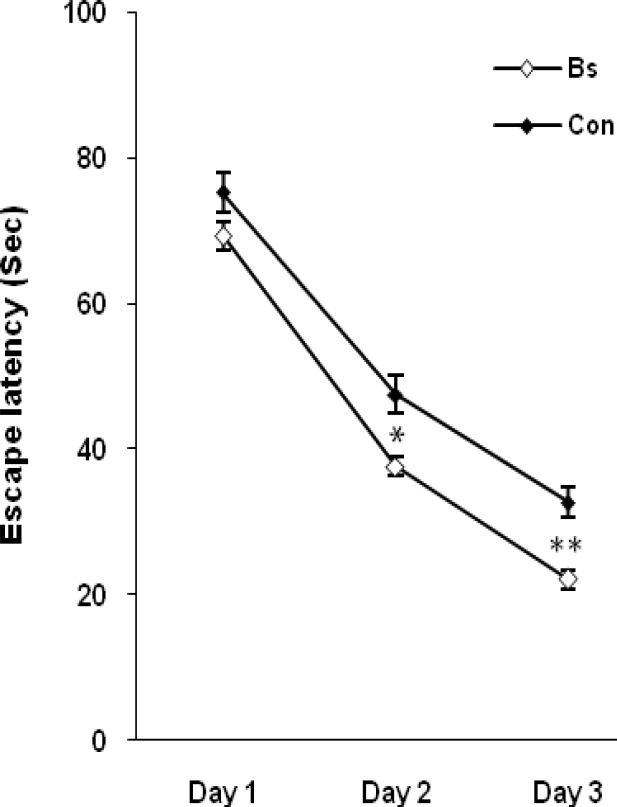
Effect of the extract of *Boswellia serrata* (Bs) on mean±SEM time to escape in the Morris water maze over the days of testing, *p= 0.05; **p = 0.01

Mean brain weights of Bs-treated rats and respective controls were 1.59±0.08 and 1.52±0.12 g, respectively (p=0.11). Chronic administration of Bs improved spatial learning capability during the three acquisition days. As shown in Figure 1, the time latency for finding the platform in the water maze test was significantly lower in Bs-treated group than the control rats. Throughout the experiment, the swim speed of the aged rats between both groups did not differ statistically (data not shown).

**Figure 2:**
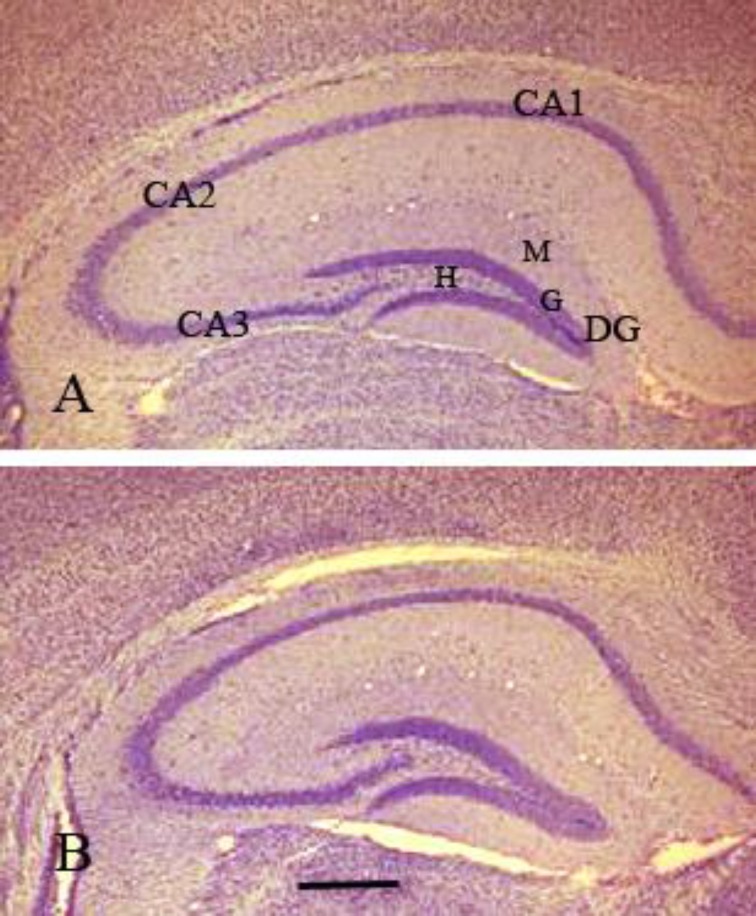
Low magnification micrograph of a coronal section through the hippocampi of control (A) and boswellia-treated (B) aged rats. Dentate gyrus (DG) is composed of molecular (M), granular (G) layers and hilus (H). Cornu ammonis (CA) is divided to CA1, CA2 and CA3. Boswellia-treated aged rats (B) display greater volumes than controls (A) in The DG and its layers. Scale bar =500 µm and applies to both frames

Statistical data (Table 1) and Qualitative observations (Figure 2) indicated that volumes of the DG and its layers were significantly greater in Bs-treated aged rats than controls.

Comparisons between two groups revealed that the total number of dendritic segments of the granular neurons was higher in the experimental group (20.9 ± 2.03) than in controls (16.5±2.00, p=0.001). Results also showed that, in these neurons, the total dendritic length was larger in Bs-treated (1522 ± 149.1 µm) than control animals (1344± 83.6 µm, p=0.011 ). 

**Table 1:** Volumes of the layers of the dentate gyrus (mm^3^) in the Bs-treated aged rats and respective controls (n=8 in each group) Value represent the mean±SD.

Dentate Gyrus	Control	Bs-treated
**Molecular**	6.13±0.67	6.84±0.71*
**Granular**	1.98±0.28	2.30±0.30*
**Hilus**	2.11±0.23	2.38±0.29*
**Whole**	10.2±1.12	11.5±1.29*

* p<0.05

**Figure 3:**
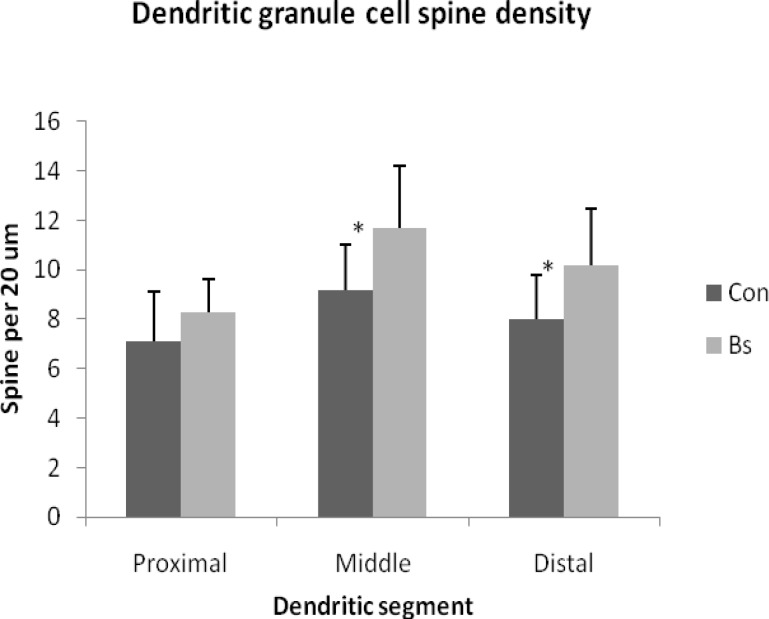
Effect of *Boswellia serrata* (Bs) treatment on the number of dendritic spines on the proximal, middle and distal 20-µm dendritic segment of dentate granule cell in aged rats. The bars represent the mean±SD.* p<0.05.

The density of spine on the middle and distal segments of dendritic tree of granule cells was significantly greater in Bs-treated than control animals. There is no significant Bs effect on the proximal dendritic spines (Figure 3). 

**Figure 4:**
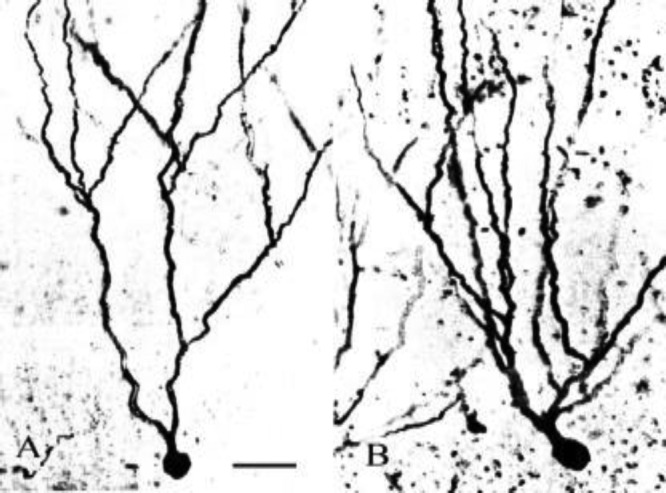
Photomontages of Golgi-impregnated dentate granule neuron from control (A) and Boswellia (Bs)-treated aged rats (B). Dentate granule cell from Bs-treated rats displays an obvious enhancement of dendritic segments as compared to similar neurons from control rats. Scale bar =30 µm and applies to both frames

Qualitative observations also revealed that an increased dendritic complexity in the dentate granule cells was present in sections from the Bs-treated aged rats as compared to the controls (Figure 4).

**Figure 5:**
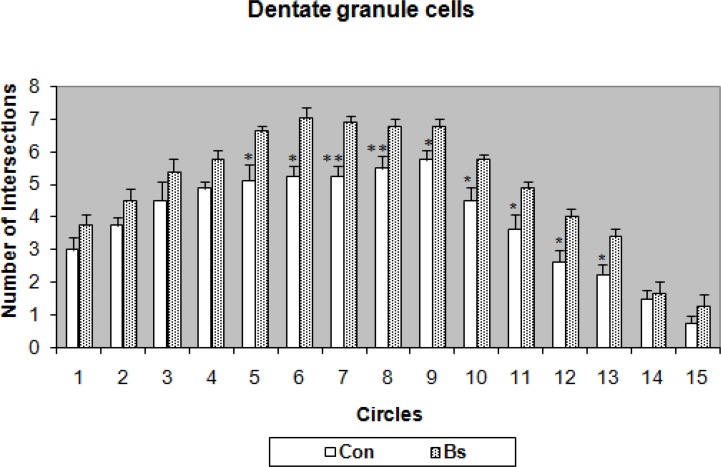
Dendritic branching density of hippocampal granule cells for Boswellia (Bs)-treated aged rats and respective controls. Vertical bars represent SEM. (* p<0.05, ** p<0.01

Study of the dendritic intersections of dentate granule cells also showed that the effect of Bs was significant for circles 5 to 13 where the intersections in the Bs group were more than the control group (Figure 5).

## Discussion

In the present experiment, treatment of aged rat with 100 mg/kg Bs resin for 8 weeks improved escape learning in the water maze. Stereological volume estimation revealed that Boswellia-treated aged rats had greater volumes than controls in the DG and its layers.

Quantitative morphological analysis of dendritic architecture of Golgi-impregnated hippocampal granule cells also indicated that Bs induced an increase in dendritic arbors associated with more dendritic spine density of these neurons in aged rat. 

In agreement with previous experiments on young rats (Farshchi et al., 2010 ▶; Hosseini et al., 2010 ▶), the result of this study showed that Boswellia-treated aged rats had better spatial learning performance than controls. In an earlier study, we evaluated the effect of total Boswellia extract administration (300mg/kg, three times a day) via gavage for 1, 2, and 4 week(s) in adult male albino Wistar rats on spatial memory parameters using Morris water maze task (Mahmoudi et al., 2011 ▶). Results showed that Boswellia-treated animals had significant decrease in escape latency and swimming distance compared to controls. Furthermore, Boswellia-treated rats spent longer time in target quarter in probe trial sessions. However, swimming speed was similar during the testing trials in all groups. Consequently, our findings indicated that Boswellia extracts improve spatial memory retention irrespective of the treatment period.

The changes in dendritic morphology of hippocampal granule cells that we observed in this study, may be involved in the improvement of hippocampal-related memory in Bs-treated rats. Differences in branching patterns and in the amount of dendritic spines have been suggested to modulate the spatial integration of synaptic to an individual neuron (Purves, 1988 ▶). Increase in these parameters could result in larger total dendritic surface area available for synaptic contact. It has to be noted that a positive influence of spatial learning tasks on dendritic spine density in hippocampal neurons was reported (Ramírez-Amaya et al., 1999 ▶; Bailey and Kandel, 2008 ▶; Butz et al., 2009 ▶) but our experiment, in which both Bs administration and behavioral tests were done in same animals, did not allow to separately measure the effects of spatial learning tests and Boswellia extract on morphology of dentate granule neurons in Bs-treated rats. However, several studies employed experiments similar to our study to evaluate the effect of different agents on dendritic spine density (Manikandan et al., 2006 ▶; Salas-Ramirez et al., 2010 ▶; Valladolid-Acebes et al., 2013 ▶).

Although identification of the precise biological mechanisms behind the Boswellia-induced structural changes of the DG and dentate granule cells were not the purpose of this study, the possible mechanisms could be: increase in the dendritic material of granule neurons may be consequences of an outgrowth of new dendritic branches or reduction of the speed of dendrite regressive changes followed by long-term Bs exposure.

*In vitro* studies showed that beta boswellic acid, the main component of Bs gum, could increase dendrite branching in hippocampal neurons (Karima et al., 2010 ▶) and also prevent microtubule proteins destabilization (Karima et al., 2012 ▶) as a cause of neurite degeneration during aging (Himeda et al., 2005 ▶). It is also reported that another component of Boswellia resin, Incensole acetate, decreases neurodegeneration through its anti-inflammatory effects on the brain (Moussaieff et al., 2008a ▶). Nemat et al. (2013) ▶ reported that *B. serrata* could ameliorate the neurodegenerative characteristics of AD (Nemat et al., 2013 ▶). 

Menon and Kar (1971) ▶ reported that ether extract of *B. serrata* resin produces analgesic and sedative effects in rats (Menon and Kar, 1971 ▶). Moussaieff et al. (2008b) ▶ also found that incensole acetate, a Boswellia resin component, causes anxiolytic and anti-depressive effects in mice.

It is reported that the granule cells of DG are rich in corticosteroid receptors and their sensitivity to stress hormones is well recognized (Reul and De Kloet, 1985 ▶). Therefore, it could be proposed that a decrease in exposure to stress following a chronic treatment with Bs has a neuroprotective effect and could lower neurotoxicity and neuronal degeneration in this region (Sapolsky, 1999 ▶). Decrease in stress hormones might lead to an increase in neurotrophins in the hippocampus (smith et al., 1995 ▶).

Furthermore, the beneficial effects of Bs on dendritic morphology of granule neurons could be related to its antioxidant activity (Mothana et al., 2009 ▶).

In conclusion, this study indicated that chronic administration of Boswellia resin in the aged Wistar rat improves learning capability along with enhancement of dendritic arbors and dendritic spines in hippocampal granule cells. We therefore propose it as a novel potential neuroprotective agent and therapeutic potential of Boswellia resin in age-associated memory decline and neurodegenerative diseases should be considered. This study also provided a neuroanatomical substrate that may be relevant to the memory improvement in BS-treated aged rats. 
